# Diversity and function of membrane glycerophospholipids generated by the remodeling pathway in mammalian cells

**DOI:** 10.1194/jlr.R046094

**Published:** 2014-05

**Authors:** Daisuke Hishikawa, Tomomi Hashidate, Takao Shimizu, Hideo Shindou

**Affiliations:** * Department of Lipid Signaling, Research Institute, National Center for Global Health and Medicine, Tokyo 162-8655, Japan; † Department of Biochemistry and Molecular Biology, Faculty of Medicine, University of Tokyo, Tokyo 113-0033, Japan; § Core Research for Evolutional Science and Technology (CREST), Japan Science and Technology Agency, 4-1-8 Honcho, Kawaguchi, Saitama 332-0012, Japan

**Keywords:** lysophospholipid acyltransferase, membrane biology, phospholipid metabolism

## Abstract

Cellular membranes are composed of numerous kinds of glycerophospholipids with different combinations of polar heads at the *sn*-3 position and acyl moieties at the *sn*-1 and *sn*-2 positions, respectively. The glycerophospholipid compositions of different cell types, organelles, and inner/outer plasma membrane leaflets are quite diverse. The acyl moieties of glycerophospholipids synthesized in the de novo pathway are subsequently remodeled by the action of phospholipases and lysophospholipid acyltransferases. This remodeling cycle contributes to the generation of membrane glycerophospholipid diversity and the production of lipid mediators such as fatty acid derivatives and lysophospholipids. Furthermore, specific glycerophospholipid transporters are also important to organize a unique glycerophospholipid composition in each organelle. Recent progress in this field contributes to understanding how and why membrane glycerophospholipid diversity is organized and maintained.

One of the major components of cellular membranes is a class of molecules known as glycerophospholipids, which are synthesized from glycerol-3-phosphate (G3P) in a de novo pathway that initially produces phosphatidic acid (PA) and diacylglycerol (DAG) or cytidine diphosphate-DAG (CDP-DAG) (1–3). Via the de novo pathway, various types of glycerophospholipids with different polar heads at the *sn*-3 position in the glycerol backbone, such as phosphatidylcholine (PC), phosphatidylethanolamine (PE), phosphatidylserine (PS), phosphatidylinositol (PI), phosphatidylglycerol (PG), and cardiolipin (CL) are generated (4, 5). Subsequently, glycerophospholipid acyl chains are remodeled by the orchestrated reactions of phospholipase As (PLAs), acyl-CoA synthases, transacylases, and lysophospholipid acyltransferases (LPLATs) (5–9). This glycerophospholipid remodeling (also called Lands’ cycle) was originally described in 1958 and is involved in the generation of a large variety of cellular glycerophospholipids (**Fig. 1**) (10, 11). Thus far, investigations of glycerophospholipid remodeling have mainly focused on PLAs, especially in the production of lipid mediators (6–8). However, in recent years, various LPLATs have been identified from the 1-acylglycerol-3-phosphate *O*-acyltransferase (AGPAT) and membrane bound *O*-acyltransferase (MBOAT) families (**Table 1**). Although studies with tissue homogenates initially suggested that each LPLAT recognizes a specific substrate, isolated LPLATs have shown promiscuous substrate specificities (5, 9, 12). Because the acyl composition of membrane glycerophospholipids is known to affect not only the production of lipid mediators but also membrane properties, characterizing these LPLATs will reveal the biological importance of membrane glycerophospholipid diversity.

**Fig. 1.**
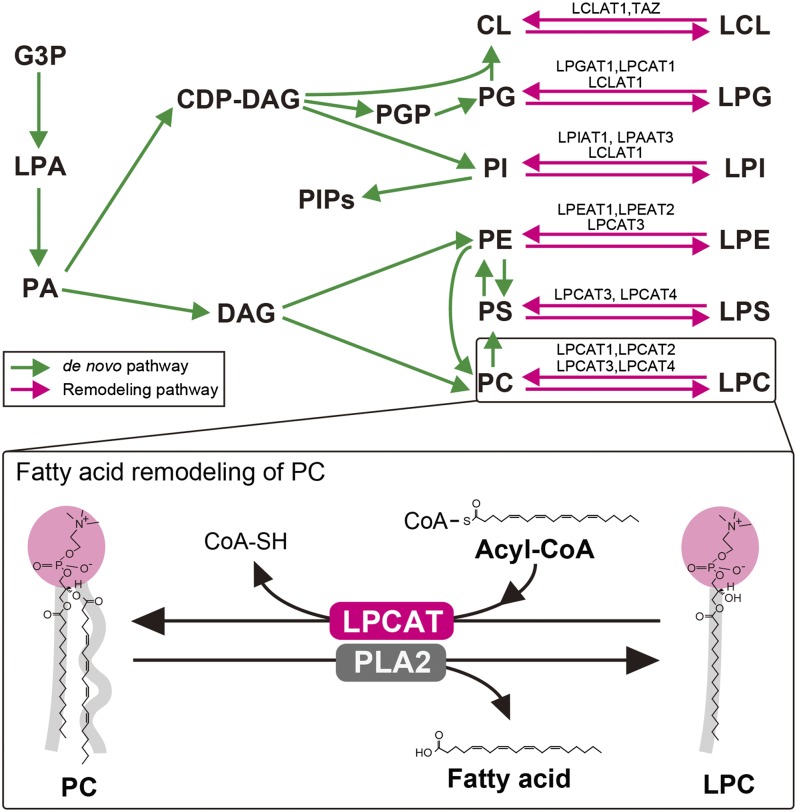
Biosynthetic pathways of glycerophospholipids. Upper panel shows the de novo synthesis (green lines) and the fatty acid remodeling (magenta lines) of glycerophospholipids. LPLATs involved in each reacylation of the lysophospholipids are indicated. Lower panel shows an example of the fatty acid remodeling of PC. In this reaction, PLA2s release fatty acid (arachidonic acid) from the *sn*-2 position of PC, while LPCATs catalyze the reacylation at the *sn*-2 position of LPC using acyl-CoA (arachidonoyl-CoA). The details are discussed in the text. PGP, phosphatidylglycerolphosphate.

**TABLE 1.** Summary of characteristics of LPLATs

		Substrate In Vitro				
Name	Other Names	Lysophospholipid	Acyl-CoA	Expression	Phenotypes of KO, Knockdown, and Mutations In Vivo	References
LPAAT1*^a^*	AGPAT1, LPAATα	LPA	—	Ubiquitous	—	(117–119)
LPAAT2*^a^*	AGPAT2, LPAATβ	LPA, LPI	—	Adipose, liver, pancreas, heart	Lipodystrophy, diabetes	(117, 120–123)
LPAAT3*^a^*	AGPAT3, LPAATγ	LPA, LPG, LPC, LPE, lyso-PAF	PUFA-CoA	Testis, adipose, liver, kidney	—	(20, 122, 124)
LPCAT1*^a^*	AGPAT9, Aytl2	LPC, lyso-PAF, LPE	Saturated species, acetyl-CoA	ATII cells in lung, retina	Reduction of DPPC in pulmonary surfactant, retinal degeneration	(32, 42–44, 46, 125, 126)
LPCAT2*^a^*	AGPAT11, Aytl1	LPC, LPS, LPE	PUFA-CoA, acetyl-CoA	Spleen, macrophage, neutrophil	—	(16, 125)
LPCAT3*^b^*	MBOAT5	LPC, LPE	PUFA-CoA	Testis, liver, kidney	Hepatic inflammation in ob/ob mouse	(17, 18, 26, 29, 127)
LPCAT4*^b^*	MBOAT2	LPE, LPS	Oleoyl-CoA	Testis, epididymis, ovary, brain	—	(17, 26)
LPEAT1*^b^*	MBOAT1	LPE	Oleoyl-CoA	Testis, epididymis, ovary, brain	Brachydactyly-syndactyly syndrome	(17, 26)
LPEAT2*^a^*	AGPAT7, Aytl3, LPCAT4	LPI	—	Brain	—	(128)
LPIAT1*^b^*	MBOAT7, MBOA7	LPI	PUFA-CoA	Ubiquitous	Postnatal lethal, atrophy of the cerebral cortex and hippocampus, altered fatty acid composition of PI and PIPs	(19, 20, 63, 65)
LPGAT1*^a^*		LPG	—	Liver, heart, small intestine, kidney	—	(45)
LCLAT1*^a^*	AGPAT8, ALCAT1, LYCAT1	LCL, LPG, LPA, LPI (2-acyl and 1-acyl)	—	Liver, heart, pancreas, kidney	Protected from obesity and insulin resistance, prevent of T4-induced cardiomyopathy, altered fatty acid composition of PI and PIPs	(5, 103–109)
TAZ*^a^*	G4.5	LCL	Transacylation	Heart, skeletal muscle	Barth syndrome, accumulation of MLCL and altered CL composition, cardiac abnormalities, impaired oxygen consumption rates during an exercise	(94, 101, 102, 129–133)

Gene names, families, substrates preferences, mRNA expression patterns, and in vivo functions of LPLATs are summarized. Please note that there are several inconsistent reports about the enzymatic substrates in vitro.

a AGPAT family member.

b MBOAT family member.

In mammalian cells, glycerophospholipid composition differs among cell types, organelles, and inner/outer membranes, and these differences are known to play important roles in various cellular functions including signal transduction, vesicle trafficking, and membrane fluidity (4). Recently, several molecules involved in phospholipid transport between membranes and in phospholipid scrambling in plasma membranes have been identified. These factors are also important for constructing the specific composition of local membranes.

In this review, we summarize and discuss the biological importance of the variety of membrane glycerophospholipids generated via glycerophospholipid remodeling by LPLATs.

## GENERATION OF PUFA-CONTAINING GLYCEROPHOSPHOLIPIDS

Glycerophospholipid remodeling by the concerted action of PLAs and LPLATs is important for the production of PUFA-containing glycerophospholipids (8). Glycerophospholipids containing PUFAs, such as arachidonic acid, linoleic acid, EPA, and DHA, are known as major sources of fatty acid-derived lipid mediators and endocannabinoids (8, 13–15). Although numerous studies have shown the importance of PLAs in producing lipid mediators, the involvement of LPLATs in lipid mediator production is poorly understood (6, 7).

At present, lyso-PC (LPC) acyltransferase (LPCAT)2, LPCAT3, lyso-PI (LPI) acyltransferase (LPIAT)1, and lyso-PA (LPA) acyltransferase (LPAAT)3 are reported to incorporate PUFAs into lysophospholipids with different acceptor preferences (16–20). LPCAT3 is ubiquitously expressed, especially in liver, testis, kidney, pancreas, and adipose tissue (17, 18). Expression of LPCAT3 mRNA is controlled by PPARα and liver X receptors, and is induced during adipogenesis (21, 22). Knockdown of LPCAT3 by siRNA reduces arachidonic acid incorporation into PC and production of eicosanoids (23). Similar results were obtained from the treatment of thimerosal, a LPLAT inhibitor, and triacsin C, an acyl-CoA synthetase inhibitor (24–28). These reports suggested that the control of arachidonic acid pools in membrane glycerophospholipids is important for the eicosanoid production. Furthermore, it has been reported that induction of LPCAT3 ameliorates saturated free fatty acid-induced endoplasmic reticulum (ER) stress in vitro (29). Using liver-specific LPCAT3 overexpression and knockdown mice, the study demonstrated that LPCAT3 regulates hepatic inflammatory cytokine levels and inflammation. Although the exact mechanism is unclear, the authors suggest that LPCAT3 may control inflammation by altering the fatty acid composition of PC.

Although LPCAT2 and LPCAT3 can produce arachidonic acid-containing PC, the substrate preference and expression pattern for each differs. While LPCAT3 prefers 1-acyl LPC as an acyl acceptor, LPCAT2 utilizes both 1-acyl LPC and 1-alkyl LPC (16–18). LPCAT2 is highly expressed in inflammatory cells such as macrophages and neutrophils, which contain ether-phospholipids, and LPCAT2 is believed to contribute to the production of lipid mediators in these cells (16). Induction of LPCAT2 has been observed in three scenarios: *i*) in macrophages by lipopolysaccharide and CpG oligodeoxynucleotide 1826 stimulation; *ii*) in the spinal cords of mice with experimental allergic encephalomyelitis; and *iii*) in mice with peripheral nerve injury (16, 30, 31). These observations support the hypothesis that LPCAT2 may be involved in lipid mediator production under inflammatory conditions. In addition to LPCAT activity, LPCAT2 and LPCAT1 also possess lyso-platelet-activating factor (PAF) acetyltransferase (lysoPAFAT) activity for the production of PAF (16, 32). It has been reported that LPCAT2, but not LPCAT1, is activated by phosphorylation at Ser34 by lipopolysaccharide stimulation for 30 min (33). The biological importance of the dual activities in PAF and PAF-precursor glycerophospholipid production remains to be elucidated.

In general, it is thought that PUFAs are mainly incorporated into glycerophospholipids in the remodeling pathway. However, DHA-containing glycerophospholipids are synthesized in the remodeling pathway as well as in the de novo pathway, because CDP-ethanolamine:DAG ethanolamine transferase and PE-*N*-methyltransferase prefer DHA-containing DAG and PE, respectively, in rat liver microsomes (34–36). In fact, LPAAT3 can produce DHA-containing PA, and LPAAT3 may contribute to the production of DHA-containing glycerophospholipids in the de novo pathway. Overexpression of LPAAT3 in HeLa cells inhibits Golgi tubule formation and protein trafficking (37). Although the mechanism underlying this is unclear, it is possible that the products of LPAAT3 (PUFA-containing PAs) may affect membrane properties. Because PUFAs are reported to increase membrane fluidity, these enzymes may be important not only for lipid mediator production, but also for cellular functions, such as signal transduction and stabilization of proteins, by controlling the biophysical properties of the membrane (38).

## FUNCTIONS OF DISATURATED GLYCEROPHOSPHOLIPIDS

Pulmonary surfactant is produced in alveolar type II (ATII) cells and is secreted into the alveolar space to prevent collapse (39). Pulmonary surfactant is composed of lipids (∼90%), mainly dipalmitoyl-PC (DPPC), and associated proteins (∼10%) (39, 40). The microsomal fraction from ATII cells exhibits high LPCAT activity with palmitoyl-CoA, indicating that surfactant DPPC is produced in the remodeling pathway (41). Indeed, LPCAT1, which is highly expressed in ATII cells, shows a preference for palmitoyl-CoA as an acyl donor (42, 43). Recently, LPCAT1 gene-trapped mice were reported to have reduced LPCAT activity, disaturated PC content in the lung, and a low survival rate (44). Pulmonary surfactant collected from dead LPCAT1 gene-trapped mice was less able to reduce surface tension than that of wild-type mice. This report indicated that LPCAT1 was important for pulmonary surfactant phospholipid production in vivo (44). PG is a second-order glycerophospholipid (∼10% of surfactant phospholipid) in pulmonary surfactant. In the remodeling pathway, both lyso-PG (LPG) acyltransferase (LPGAT)1 and LPCAT1 are reported to have LPGAT activities in vitro (42, 45). Further studies are needed to clarify the mechanisms underlying high-level PG production in the lung.

Linkage analysis in mice has shown that LPCAT1 is mutated in rd11 (one nucleotide insertion) and B6-JR2845 (seven nucleotide deletion) mice, which exhibit retinal degeneration (46). Because disaturated PC is abundant in disk membranes of rod outer segments (47), LPCAT1 may have important roles for function of the disk membrane.

Membrane fatty acid saturation of glycerophospholipids by stearoyl-CoA desaturase 1 knockdown and palmitic acid treatment were reported to induce the ER stress (48–51). Although it is unclear whether glycerophospholipid remodeling is involved in this cellular response, LPCAT1 may also contribute to regulate the level of saturated fatty acid in glycerophospholipids.

Moreover, recent studies suggest a correlation between LPCAT1 expression and cancer progression (52–54). Because LPCAT1 has both LPLAT and lysoPAFAT activities, further studies are needed to determine which LPCAT1 products, disaturated glycerophospholipids or PAF, are involved in cancer progression.

## GLYCEROPHOSPHOLIPIDS AS SIGNALING MOLECULES

Of the cellular membrane glycerophospholipids, PS and PI phosphates (PIPs) act as signaling molecules via interactions with specific proteins (55, 56). Thus, although their percentage of total cellular glycerophospholipids is low, PS and PIPs play important roles in various cellular functions. PIPs can be recognized by various binding domains, such as the pleckstrin homology, Fab1/YOTB/Vac1/EEA1, phox homology, and epsin *N*-terminal homology domains (56–58). On the other hand, γ-carboxyglutamic acid, protein kinase C C2, discoidin C2, and kinase associated-1 are reported to be PS-recognizing domains (57, 59, 60). Exceptionally, the pleckstrin homology domain of evectin-2 is reported to bind PS but not PIPs (61).

PIPs are biosynthesized by the reversible phosphorylation of three of the five hydroxyl groups on the inositol head group of PI (56). Arachidonic acid is the most predominant acyl chain found in the *sn*-2 position of PI and PIPs (62, 63). LPIAT1 prefers arachidonoyl-CoA as an acyl donor and generates arachidonic acid-containing PI. Because acyltransferase activities for lyso-PIPs are very low, the enrichment of arachidonic acid in PI and PIPs seems to be controlled in the PI remodeling pathway (19, 26). Recently, the phenotype of LPIAT1 KO mice was reported by two different groups (63, 65). LPIAT1 KO mice were postnatal lethal and showed atrophy of the cerebral cortex and hippocampus. LPIAT1 deficiency caused abnormal cortical lamination and delayed neuronal migration in the cortex at embryonic day 18.5 (65). Fatty acid compositions and the cellular amounts of PI and PIPs were also changed in LPIAT1 KO mice (63, 65). Further studies are needed to clarify whether the reduction or the altered fatty acid compositions of PI and PIPs contributed to the phenotypes of LPIAT1 KO mice. LPIAT1 KO mice showed an almost complete loss of LPIAT activity with arachidonoyl-CoA in brain, liver, kidney, and testis (65). In the brains of LPIAT1 KO mice, 65% of the normal level of arachidonic acid-containing PI was present (63, 65). Thus, de novo synthesis also seems to be important for the incorporation of arachidonic acid into PI. On the other hand, it is reported that exogenously supplied palmitoleate (16:1) was preferentially incorporated into PI and induced cell proliferation (66). In addition, a difference between the fatty acid composition of PIPs of whole cell membrane fractions and that of nuclear membrane fractions has also been reported, indicating that the acyl chains of PI and PIPs may have some specific functions (67). Furthermore, it has been reported that LPIAT1 mainly localizes at mitochondria-associated membranes (MAMs), where acyl-CoA synthetase long-chain 4 is expressed, and interacts with the small subunit of serine palmitoyl-transferase a (68). This report suggests that the specific localization of LPLATs through interactions with other related proteins may also be important for substrate recognition. Although LPAAT3 also has LPIAT activity with PUFA-CoA in vitro, little information concerning its biological roles is available (20).

PS is highly enriched in the inner leaflet of the plasma membrane and in intracellular organelles such as recycling endosomes, and acts as a tag for the recognition of apoptotic cells, coagulation, and vesicle trafficking by PS-binding proteins (59). It is known that PS in the plasma membrane is exposed to the outer leaflet during platelet activation and apoptosis by the action of Ca^2+^-dependent phospholipid scramblases (69). A recent study identified TMEM16F and Xkr8 as the key molecules for PS exposure in this process (70–72). Furthermore, binding of evectin-2 to PS in the recycling endosomes is essential for retrograde membrane trafficking (61, 73). While the mechanisms underlying the transport of PS from the ER to the specific organelle are unknown, yeast oxysterol-binding homology (Osh)6, Osh7, human oxysterol-binding protein related protein (ORP)5, and ORP10 have been reported to bind and transport a single PS molecule between membranes (74). Because the acyl-chain composition of PS purified with Osh6 is limited when compared with yeast PS, the acyl-chain composition of PS may also be important for ligand recognition by PS transporters (74, 75). This finding suggests that not only polar heads, but also fatty acid compositions contribute to PS transport. LPCAT3 and lyso-PE (LPE) acyltransferase (LPEAT)1 have been reported to possess lyso-PS (LPS) acyltransferase (LPSAT) activities with arachidonoyl-CoA and oleoyl-CoA, respectively (17, 18). Further studies are required to elucidate the roles of PS fatty acid composition in intracellular transport and other cellular functions.

## CONE-SHAPED GLYCEROPHOSPHOLIPIDS AND MEMBRANE CURVATURE SENSORS

Cone-shaped glycerophospholipids with small polar heads (PE, PA, and CL) and/or bulky acyl chains (monounsaturated fatty acid-containing glycerophospholipids) are known to have important roles in membrane fusion and fission steps during endocytosis, exocytosis, cytokinesis, and vesicle trafficking (76–78). In the curved membrane, cone-shaped glycerophospholipids provide loosely packed regions, termed lipid-packing defects, which are recognized by membrane curvature sensors possessing amphipathic lipid-packing sensor motifs. They consist of an α-helix of 20 to 40 amino acids with a serine- or threonine-rich polar face (79). Membrane curvature sensors containing amphipathic lipid-packing sensor motifs are important for vesicle and lipid trafficking (80). Recently, we reported that the Sec14 domain of Sec14-like 3 also senses lipid-packing defects in liposomes (81). These reports suggest that cone-shaped glycerophospholipids are important for various cellular functions, such as lipid transport.

LPEAT1 and LPCAT4 are reported to prefer LPE and oleoyl-CoA as substrates (17) and produce cone-shaped glycerophospholipids. Although the cellular functions of these enzymes are unclear, regulation of cone-shaped glycerophospholipid biosynthesis by LPEAT1 and/or LPCAT4 may affect vesicle trafficking, membrane fusion, and endocytosis/exocytosis by providing the appropriate lipid-packing defects on curved membranes. Several reports showed that inhibition of LPCAT and LPEAT activities by a broad LPLAT inhibitor, CI-976 (2,2-methyl-*N*-(2,4,6,-trimethoxyphenyl)dodecanamide) enhanced Golgi tubulation and membrane trafficking (82). Several types of PLAs were also reported to be important in intracellular membrane trafficking and fusion events (83). The regulation of membrane glycerophospholipid composition in the remodeling pathway affects the cellular membrane functions.

Disruption of the LPEAT1 gene was reported in a patient with a brachydactyly-syndactyly syndrome (84). Thus, the cone-shaped glycerophospholipids produced by LPEAT1 may be important for normal organogenesis.

## GLYCEROPHOSPHOLIPID METABOLISM AND FUNCTION IN MITOCHONDRIA

Mitochondria are dynamic organelles involved in crucial cellular processes, such as cell respiration and energy production. CL is a major glycerophospholipid in mitochondria, especially in the inner membrane, which affects the stability and activity of various membrane protein complexes and metabolite carriers (85, 86). CL is a unique dimeric glycerophospholipid possessing two PAs, bridged by a glycerol, and four fatty acyl chains. Although the molecular mechanism of CL synthesis is not completely understood, recent studies have identified new molecules related to the process, such as a protein that transports PA from the outer membrane to the inner membrane (87, 88), a mitochondrial-type CDP-DAG synthase (89), and a mammalian phosphatidylglycerolphosphate synthase (90–92). The acyl chains of CL are highly enriched with linoleic acid in the remodeling pathway (93). Tafazzin (TAZ) and lyso-CL (LCL) acyltransferase 1 (LCLAT1; also known as acyl-CoA:LCLAT1) were reported to remodel the acyl chains of CL by transacylation of CL and acylation of LCL, respectively (94–97).

Abnormal CL remodeling is observed in many pathological situations, such as aging, heart failure, and Barth syndrome (98). Mitochondria from patients with Barth syndrome exhibited lower CL content and abnormal acyl-chain compositions (99). TAZ gene mutations are responsible for Barth syndrome (100, 101). Indeed, cardiac muscle from TAZ gene knockdown mice showed an accumulation of mono-LCL and decreased tetralinoleoyl-CL (102). These observations indicate that CL acyl-chain remodeling by TAZ may be critical for CL maturation and mitochondrial functions.

In addition to TAZ, LCLAT1 is also reported to be involved in CL acyl-chain remodeling (96, 97). Whereas TAZ is localized to mitochondria, LCLAT1 is localized to the ER and MAM (97, 103). A recent study showed that insulin resistance induced by a high fat diet in LCLAT1 KO mice was improved (103). Furthermore, LCLAT1 overexpression in C2C12 cells leads to a reduction in the levels of linoleic and oleic acids and a slight increase in the levels of arachidonic acid and DHA in CL (103). Based on these results, it was suggested that the activation of LCLAT1 may be involved in the oxidative stress-induced inhibition of mitochondrial function through PUFA incorporation in CL. However, the acyltransferase activities of LCLAT1 for other lysophospholipids, such as LPA (104), LPI, LPG (105), bis(monoacylglycero)phosphate (106), and 2-acyl-LPI (5, 107–109) have also been reported. Indeed, LCLAT1 KO mice showed decreased acyltransferase activities for 2-acyl-LPI and altered composition of PI without obvious changes in other glycerophospholipid acyl species (109). Thus, more information is needed to determine the biochemical and physiological properties of LCLAT1.

Recently, the involvement of mitochondrial G3P acyltransferase (GPAT) in mitochondrial fusion in *Caenorhabditis elegans* and HeLa cells was reported (110). Because LPA supplementation and LPAAT inhibition rescued mitochondrial fragmentation in *GPAT* mutated *C. elegans*, accumulation of LPA in mitochondria seems to be important for mitochondrial fusion (110). Moreover, LCLAT1 is also reported to have a role in mitochondrial fusion (111). These results suggest that the glycerophospholipid composition of mitochondria is important for protein complex formation as well as for fusion.

## CONCLUSIONS

**Fig. 2.**
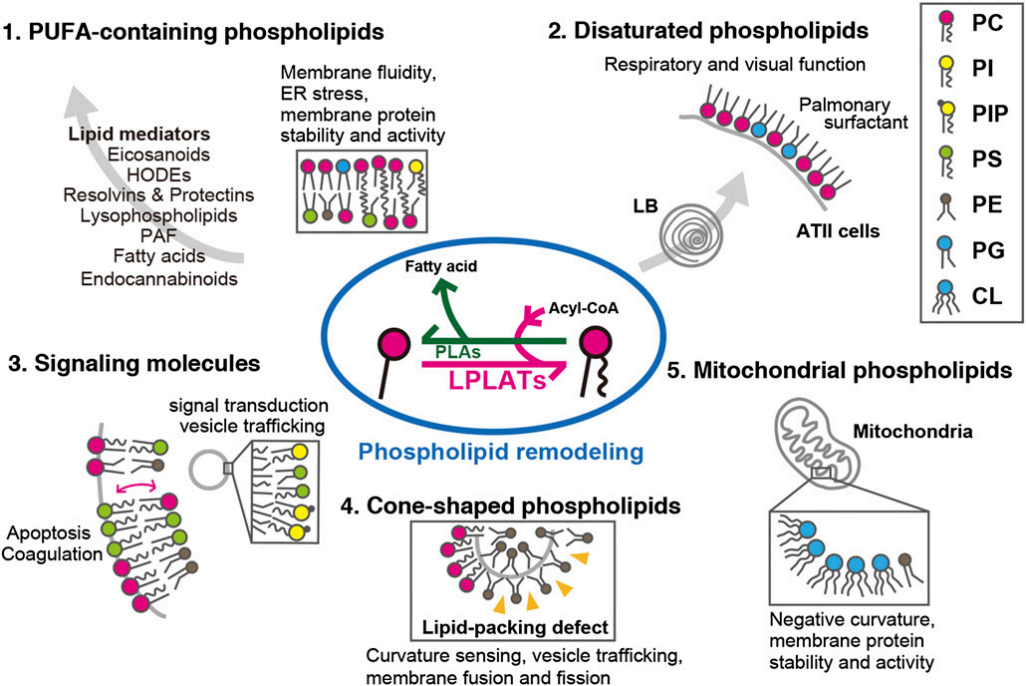
Cellular functions of glycerophospholipid remodeling and diversity. Roles of various glycerophospholipids in mammalian cells are shown. The membrane glycerophospholipid diversity produced in the fatty acid remodeling pathway may affect various cellular functions. The details are discussed in the text. LB, lamellar body.

Recent progress in LPLAT research has opened the door to understanding the contribution of membrane glycerophospholipid diversity to various cellular functions (**Fig. 2**). Moreover, the phenotype of LPLAT KO mice also has wide-ranging implications for the importance of membrane glycerophospholipids in various cellular processes (Table 1). However, the biological significance of: *i*) a single enzyme recognizing multiple substrates; *ii*) the accumulation of substrates in specific regions, such as MAMs; and *iii*) the fact that structurally dissimilar AGPAT and MBOAT family proteins can recognize the same substrate (lysophospholipids and acyl-CoAs) is as yet unknown. The substrate discrimination of LPLAT may be controlled by interactions with other proteins. Furthermore, the recent identification of the unique membrane glycerophospholipid remodeling enzymes, such as comparative gene identification 58 (CGI58), adiponutrin, cytosolic PLA2γ, and phospholipase A/acyltransferases suggest that membrane glycerophospholipid diversity is formed and maintained in many distinct ways (112–116). A more comprehensive understanding of the mechanisms and importance of membrane glycerophospholipid diversity remains to be explored in future studies.
